# Effect of *Curcumin* gel on inflammatory and anti-inflammatory biomarkers in experimental induced periodontitis in rats: a biochemical and immunological study

**DOI:** 10.3389/fmicb.2023.1274189

**Published:** 2023-11-14

**Authors:** Chenar Anwar Mohammad, Khadeeja Mohammed Ali, Aram Mohammed Sha, Sarhang Sarwat Gul

**Affiliations:** ^1^Periodontics Department, College of Dentistry, Hawler Medical University, Erbil, Iraq; ^2^Department of Periodontics, College of Dentistry, University of Sulaimani, Sulaymaniyah, Iraq; ^3^Smart Health Tower, Sulaymaniyah, Kurdistan, Iraq; ^4^Medical Laboratory Department, College of Health and Medical Technology, Sulaimani Polytechnic University, Sulaymaniyah, Iraq

**Keywords:** *Curcumin* gel, experimental periodontitis, inflammatory and anti-inflammatory biomarkers, metalloproteinase-8, C-reactive protein, alkaline phosphatase

## Abstract

This study aimed to determine the effect of local application of curcumin gels as adjunct to scaling and root planing (SRP) on the inflammatory biomarkers matrix metalloproteinase-8 (MMP-8), interleukin-6 (IL-6), C-reactive protein (CRP), and alkaline phosphatase (ALP), and the anti-inflammatory biomarker interleukin-10 (IL-10) in rats with experimentally induced periodontitis. Fifty-five adult Wistar rats with experimentally induced periodontitis were randomly divided into four groups: 15 rats received SRP + curcumin gel (CU), 15 rats received SRP + Tetracycline gel (Tet), 15 rats were treated with SRP alone, and 5 rats had experimental periodontitis without treatment (EP). Five systemically healthy rats without experimental periodontitis were used as the controls. Blood samples were collected by cardiac puncture from all groups after 2, 4, and 6 weeks of therapy. Biomarker levels determined by enzyme-linked immunosorbent assay (ELISA) and, ANOVA were used to compare the study groups. The results showed a significant increase in pro-inflammatory biomarkers and a significant decrease in anti-inflammatory biomarkers in the EP group compared with the control group (*p* < 0.05). The local application of curcumin or tetracycline gels resulted in a significant reduction in all inflammatory biomarkers at all periods of examination compared to the EP group. IL-10 levels gradually increased after 2 weeks, peaked at 4 weeks, and then decreased after 6 weeks, however, Tet showed statistically significant improvement compared to CU (*p* < 0.05). Adjunctive application of CU gel was as effective as Tet gel in the treatment of EP in rats by reducing inflammatory biomarkers and enhancing anti-inflammatory cytokines.

## Introduction

Periodontal diseases are chronic inflammatory diseases that affect the supporting tissues of the teeth. The advanced form of the disease, called periodontitis, is characterized by loss of periodontal attachment apparatus that can not be reverted (Hernández-Monjaraz et al., 2018). The main etiological factor in the initiation of periodontal diseases is dental plaque. Periodontal pathogens induce and immunoinflammatory host response resulting in tissue destruction (Hernández-Monjaraz et al., 2018). The chronic inflammation caused by periodontitis can further destroy tooth-supporting tissues may resulting in tooth loss in advanced form of the disease. Complex interactions between inflammatory and tissue remodeling mediators are involved in the etiopathogenesis of periodontal disease (Graves, 2008).

The main constituent of the outer membrane of Gram-negative microorganisms is lipopolysaccharide (LPS), which plays a vital role in the release of inflammatory cytokines by the host, such as interleukin-6 (IL-6), interleukin-1β (IL-1β) and tumor necrosis factor alpha (TNF-α), to further promote inflammation. Consequently, the activities of nuclear factor kappa-B (NF-κB) and activator protein 1 are increased, resulting in periodontal attachment loss and alveolar bone resorption (Zhang et al., 2008).

The gold standard treatment of periodontal diseases is scaling and root surface planing (SRP) (Kim et al., 2015). Significant reduction in the load of pathogenic microbiota is nonetheless impossible as some pathogens reside in soft tissue or are located in anatomically difficult areas, such as the furcation area of teeth. As a result, antimicrobial medications are frequently utilized as adjuncts to SRP for the treatment of periodontal disease (Page, 2004). Chlorhexidine, Amoxicillin, Clindamycin, Clavulanate, Metronidazole, Azithromycin, and Tetracycline antimicrobials are widely used as adjuncts to periodontal therapy. A combination of systemic Amoxicillin and Metronidazole has been successfully demonstrated to enhance clinical periodontal parameters and significantly reduce the amount of periodontal bacteria (Zhang et al., 2008). However, several drawbacks of systemic antimicrobial medications have been reported, such as tooth discoloration, changed taste perception, and antibiotic resistance; therefore, local application of antibiotics was advised to reduce the side effects of systemic antibiotics (Matesanz-Pérez et al., 2013; Jepsen and Jepsen, 2016). On the other hand, herbal medicine is advised as an adjunctive agent to SRP as alternative to antibiotics (Mohsen et al., 2020).

Since ancient times, turmeric, also known *as Curcuma longa*, has been used as a condiment and dye. It is a traditional spice from Southeast Asia. The use of turmeric increased when it was discovered to have medicinal properties and benefits in the treatment of different diseases. The dried rhizome of *C. longa* is highly rich in phenolics, with structures that have been recognized as Curcuminoids, which are chemically related to its main component, *Curcumin* (CU), causing turmeric to be of great value in medicine. The rhizomes of *C. longa* are used for extraction of CU, a naturally occurring yellow pigment in turmeric (Stabholz et al., 1993). It constitutes approximately 3–4% of the composition of turmeric and has antibacterial, anti-inflammatory, antioxidant, and anticancer properties, with a potential role in the treatment of periodontitis (Zhang et al., 2008; Fuloria et al., 2022).

There is an increasing interest in the adjunctive use of natural substances that could result in similar improvements as antibiotics in the non-surgical treatment of periodontitis (experimentally induced or not) (Mohammad et al., 2022). Furthermore, *CU* has shown to intervene the pathophysiological process of inflammation rather than working solely as an antimicrobial substance (Livada et al., 2017). It has been reported that the anti-inflammatory actions of *CU* could be associated with inhibiting the mRNA and protein expression of cyclo-oxygenase-2 via down regulation of NF-κB activation (Plummer et al., 1999), and reducing the activity of phospholipase and lipoxygenase (Yamamoto et al., 1997; Began et al., 1998). However, further studies have been suggested to provide concrete evidence on the beneficial effects of *CU* on reduction of probing pocket depth and clinical attachment loss (Oliveira et al., 2021; Zhang et al., 2022). It is important to acknowledge that the effects of locally administered *CU* gel on the level inflammatory biomarkers of IL-6, MMP-8, CRP, ALP, and IL-10 in blood in comparison to Tetracycline have not yet been investigated. Therefore, the purposes of this study are to investigate the effect of locally administered *CU* gel after SRP in systemic inflammatory biomarkers levels and compared to Tetracycline gel in combination with SRP on the systemic inflammatory biomarkers of IL-6, MMP-8, CRP, ALP, and IL-10 in experimentally induced periodontitis (EP) in rats. The null hypothesis is that *CU* gel does not modifies systemic inflammatory markers levels compared to SRP alone (standard treatment).

## Materials and methods

### Rats and housing

The study involved fifty-five male Wistar rats weighting 250–300 g, 4–6 weeks old, maintained in the animal house of the College of Medicine, Hawler Medical University, Erbil, Iraq. Prior to initiation of the study, the rats were allowed a week to adjust to the conditions in the animal house. Five rats were housed in each cage on a 12-h light/dark cycle at an average temperature of 20 ± 5°C and a humidity level of 20–30%. Standard rodent chow and unlimited access to water were provided.

The Ethical and Animal Care Committee at the College of Dentistry/Hawler Medical University, Erbil, Iraq approved the study (approval no. 026, February 28, 2019), and all experimental procedures were completed in accordance with the Guidelines for Animal Experiments of Hawler Medical University and ARRIVE guidelines. In total, 50 rats with experimentally induced periodontitis and 5 rats as intact healthy controls were used in this study.

### *Curcumin* gel composition and preparation

In SRP + CU group, rats received 12.5 μg/mL of CU gel immediately after SRP (Kunnumakkara et al., 2017). CU gel contained CU powder: 95% CU (Bulk Supplements Pure CU 95% Natural Turmeric Extract Powder), Potassium sorbate (Analitik Kimya Ve Lab. Cih. San. Tic. Ltd. Sti. Istanbul, Turkey), Propylene glycol (Pharmaco-Aaper, Bengaluru, India), Metalose 90SH 10,000 (Shin-Etsu Chemical Co., Chiyoda, Japan) and purified water. The muco-adhesive gel was prepared by Awa Medica Company, Hawler, Kurdistan Region, Iraq.

### Induction of experimentally induced periodontitis in rats

**Figure 1:**
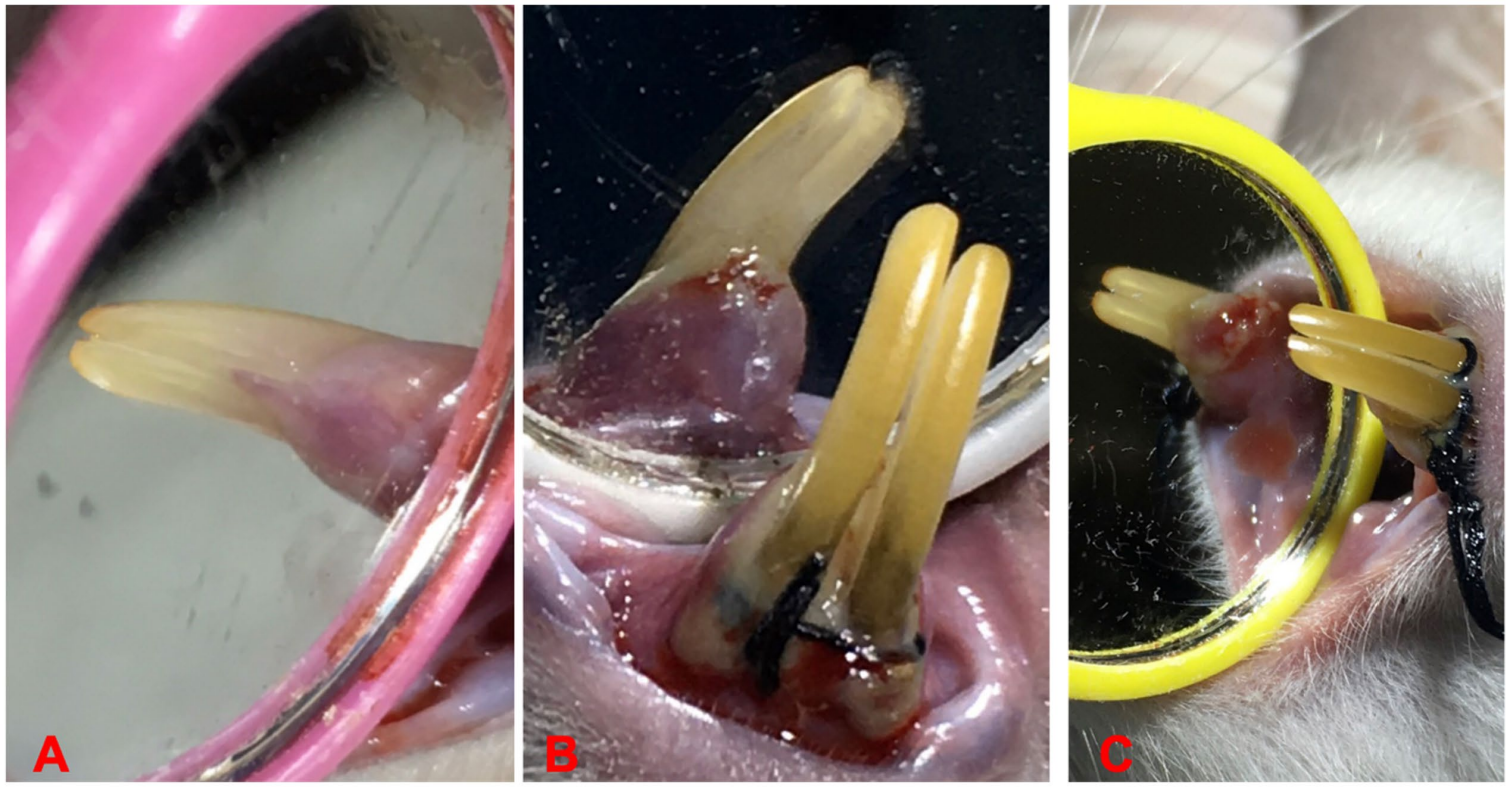
Ligature placements around cervix of lower incisors for EP induction, **(A)** before ligation, **(B)** induction of periodontitis, and **(C)** ligature induced periodontitis.

A combination of ligation and administration of revised *Porphyromonas gingivalis* was used to induce EP. The clinically isolated *P. gingivalis* from our previous study were used (Sha and Garib, 2019). An intramuscular injection of 0.12 mL/100 g body weight of Ketamine 10% and Xylazine 2% (2:1) solution was carried out to anesthetize the rats. The cervix of the upper left incisor was encircled with sterilized nylon 3–0 thread ligatures, which were then tied on the labial side of the tooth. This resulted in subgingival positioning on the palatal side and supragingival positioning on the labial side, (Iova et al., 2021), as shown in Figures 1A–C. All ligatures were checked daily, and if any were missing, they were instantly replaced. Additionally, 0.03 mL (1 × 10^6^ CFU/mL) of *P. gingivalis* was applied every 3 days to the base of the gingival sulcus of the examined tooth (upper left incisor) for a period of 10 days (Sha et al., 2021). The induction of periodontitis confirmed by presence of probing pocket depth >0.5 mm using Williams periodontal probe (Fischer and Klinge, 1994).

### Experimental design

**Figure 2:**
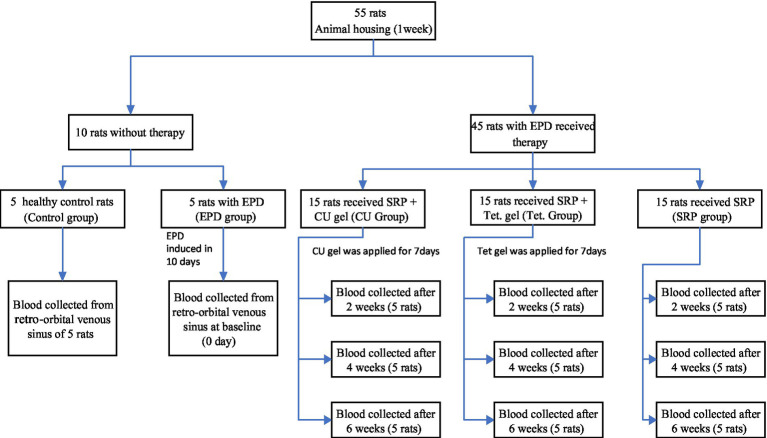
Experimental design of the study.

The 55 rats were randomly divided into the following five groups (Figure 2).

The first group included five healthy rats without ligature and inoculation with *P. gingivalis* (control group).

The second group included 5 rats with experimentally induced periodontitis without treatment (experimentally induced periodontitis (EP) group).

The third group included 15 rats that received local application of prepared *Curcumin* gel (12.5 μg/mL) into the periodontal pocket after SRP for 7 days [*Curcumin* (CU) group].

The fourth group included 15 rats that received local application of prepared Tetracycline gel (6 μg/mL) into the periodontal pocket for 7 days after SRP [Tetracycline (Tet) group].

The fifth group included 15 rats that received SRP alone (SRP group).

After induction of periodontitis for 10 days, the ligatures were removed. Rats in the CU and Tet groups then received *Curcumin* and tetracycline gels, respectively, immediately after scaling and root planing. The treatment included local administration of the gel into the pocket, twice daily, 24 h after ligature removal, and was repeated continuously for 7 days using a plastic syringe with a blunt end. The choice of dosage was based on data from other previous studies as 12.5 μg/mL for *Curcumin* gel and 6 μg/mL for Tetracycline gel (Sha and Garib, 2019; Mohammad et al., 2022). Five animals from each treated group were anesthetized and blood was withdrawn by cardiac puncture after 2, 4, and 6 weeks of therapy.

### Blood sample collection

Ketamine and Xylazine were injected intraperitoneally to euthanize the animals (100 mg/kg Ketamine and 10 mg/kg Xylazine; Park et al., 2016), and cardiac puncture was used to collect a 5-ml blood sample from each animal. Later, the blood sample was added to a gel tube and centrifuged for 10 min at 3,000 rpm to dissociate into serum and stored at −20°C for analysis of MMP-8, IL-6, IL-10, CRP, and ALP levels. Blood samples were collected from the control and EP groups (at baseline after ligature-induced periodontitis was established), and from the three treated groups after 2, 4, and 6 weeks of therapy.

### Determination of biomarker levels

Serum IL-6, IL-10, and MMP-8 concentrations were determined using rat-specific enzyme-linked immunosorbent assay (ELISA) kits according to the manufacturer’s instructions. The Rat IL-10 ELISA kit-MyBioSource (MBS355232), MMP-8 ELISA kit-MyBioSource (MBS2501356), and Rat Il-6 ELISA kit (Wuhan Fine Biotech, ER0042) was used for determination of the IL-10, MMP8, and IL-6 level, respectively. For biochemical markers, the colorimetric method (02160, BIOLABO, France) and an enzyme immunoassay determination kit (LDN, United States) were used to determine the levels of ALP and highly sensitive CRP.

### Statistical analysis

Data are presented as the mean ± standard deviation, and are normally distributed. Comparisons between the EP group at baseline (0 days) and the control healthy group and between the periodontitis EP group at baseline (0 days) and the different treatment groups at 2, 4, and 6 weeks of therapy were performed by ANOVA *post hoc* Tukey. Comparisons between the three treatment groups at 2, 4, and 6 weeks of therapy were performed using one-way ANOVA test (*F*-test), accompanied by a *post hoc* test (Tukey test). The SPSS software package (version 22; SPSS Inc., Chicago, IL, United States) was used to analyze the data.

## Results

**Figure 3:**
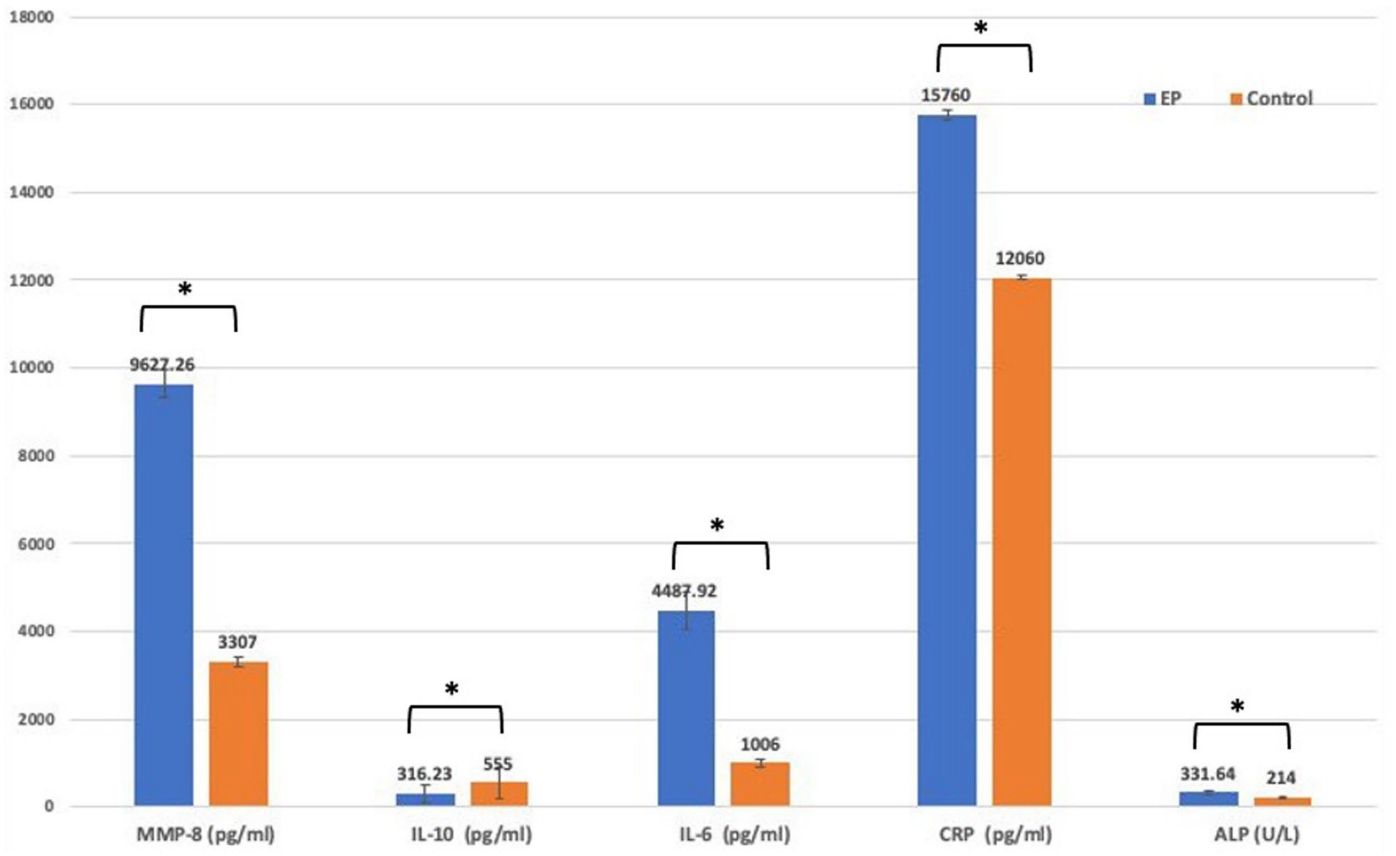
Comparison of biomarker level between EP and control group using *t*-test. EP, experimental periodontitis; MMP, matrix metalloproteinase; Il-10, interlukine10; CRP, C-reactive protein; ALP, alkaline phosphatase enzyme.

Mean serum concentrations of MM-8, IL-6, CRP, and ALP were significantly higher in the EP group than in the healthy control group. In contrast to the control group, IL-10 concentrations were significantly reduced in the EP group (Figure 3).

**Figure 4:**
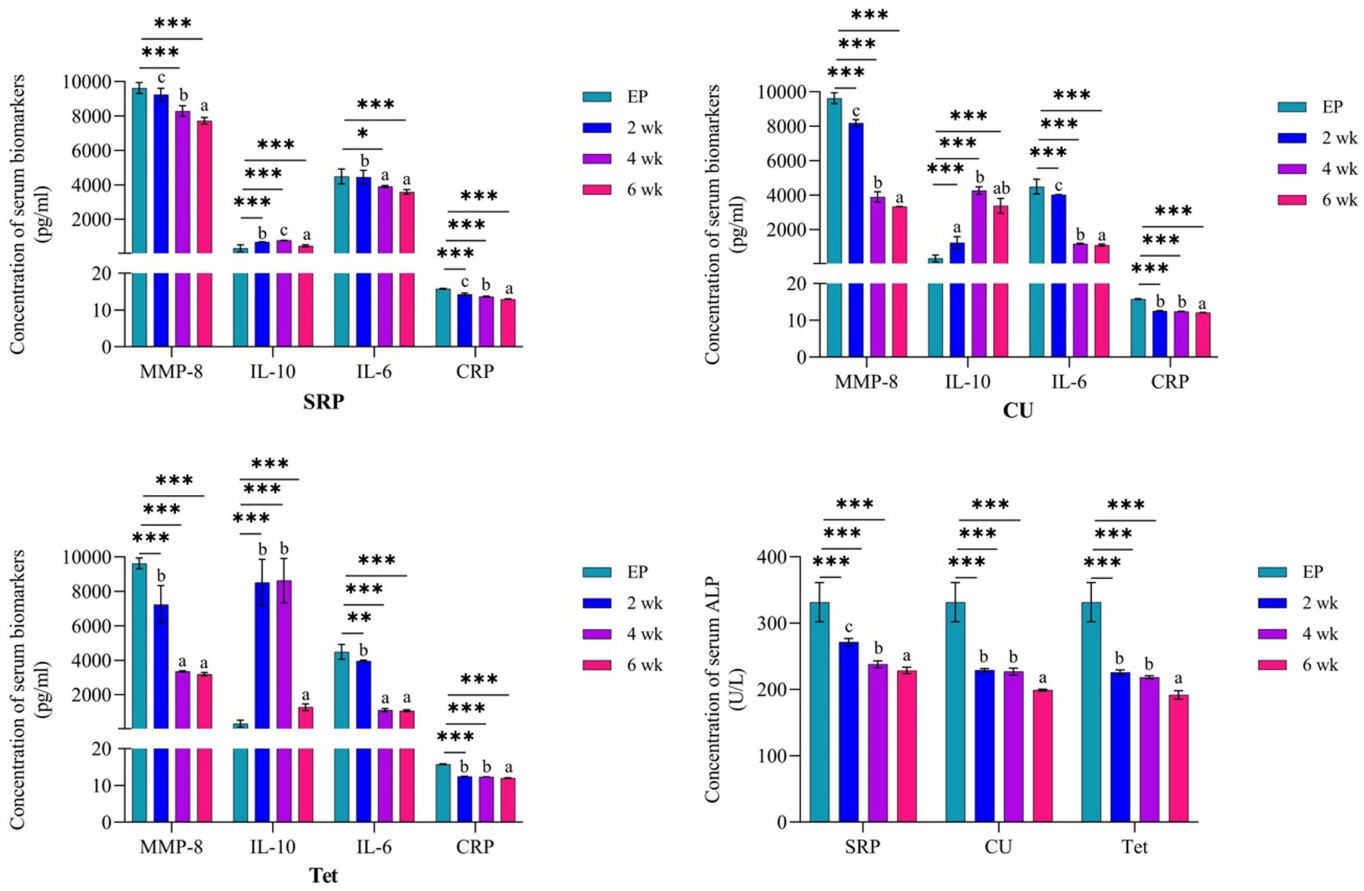
Comparison between the EP group (0 day) and three treated groups at the three different time points and the three different time points for each group using one-way ANOVA and Tukey’s test for variable time comparison. EP, experimental periodontitis; SD, standard deviation; CU, *Curcumin*; Tet, tetracycline; SRP, scaling and root planing; MMP-8, matrix metalloproteinase-8; Il-10, interlukine10; CRP, C-reactive protein; ALP, alkaline phosphatase enzyme; *p*, probability; by ANOVA *post hoc* Tukey compares 2-, 4- and 6-weeks data to base line data.

The comparison between EP at baseline (0 day) and the three treatment groups at 2, 4, and 6 weeks of therapy is shown in Figure 4. In the CU group, there was a significant reduction in the levels of MMP-8, IL-6, CRP, and ALP after 2, 4, and 6 weeks of therapy compared to EP group (*p* < 0.001). A gradual increase in IL-10 levels was observed after 2 weeks of therapy; however, with no significant differences compared to EP (*p* = 0.465), and further increasing at 4 weeks (*p* < 0.001), followed by a slight decrease after 6 weeks, but levels remained significantly higher than those in the EP (0 days) (*p* = 0.002). Similar results were found in both Tet and SRP groups (*p* < 0.001), except for the reduction in MMP-8 levels after 6 weeks of therapy in the Tet group (*p* = 0.330) and IL-6 after 2 weeks in the SRP group (*p* = 0.994), which were not statistically significant.

It is important to acknowledge that levels of examined biomarkers in all studied groups after 6 weeks of therapy were significantly reduced, and their levels were comparable to the healthy control group.

Figure 4 also shows a gradual and significant elevation in serum IL-10 levels after 2 and 4 weeks of therapy in both the Tet and the SRP groups (*p* < 0.001). The levels later slightly decreased, but with non-significant differences from EP (0 days) after 6 weeks of therapy in the Tet group (*p* = 0.286) and the SRP group (*p* = 0.138). However, the level of IL-10 in CU group at 6 week was statistically significantly higher compared to EP (*p* < 0.05).

In addition, intra-group analysis of mean serum levels of IL-6, MMP-8, IL10, CRP, and ALP at the different treatment groups was analyzed by one-way ANOVA *post hoc* Tukey’s test (Figure 4). MMP-8 and IL-6 levels in the CU group showed significant differences between time periods of 4 and 6 weeks compared to 2 weeks, whereas IL-10 levels showed non-significant differences between time points of 2 and 6 weeks and of 4 and 6 weeks. Furthermore, between 2 and 4 weeks, there were no significant changes in the CRP and ALP levels. In the Tet group, the MMP-8 and IL-6 levels at 4 and 6 weeks and the IL-10, CRP and ALP levels at 2 and 4 weeks were not significantly different. Finally, IL-6 levels in the SRP group between weeks 4 and 6 were not statistically significant (Figure 4).

## Discussion

In the current experimental study, in addition to inoculation of *P. gingivalis*, a ligature induced periodontitis model was selected because the subgingival placement of the ligature favors accumulation of biofilms around the marginal gingiva and promotes sulcular epithelial ulceration, which cause exposure of the underlining connective tissue. Invasion by periodontal pathogens into this tissue results in intense host–microbe interactions inducing chemotaxis and recruitment of inflammatory cells (Graves et al., 2012; Lima et al., 2017). Furthermore, this model is a highly predictable technique for the evaluation of adjunctive agents in periodontal therapy (Demirer et al., 2012; Kim et al., 2012).

The main focus of the present study was to compare the effectiveness of CU and Tet gels on the levels of inflammatory biomarkers, specifically IL-6, MMP-8, ALP, and CRP. The rationale behind selecting these biomarkers was associated with promising results for their use to aid in the diagnosis of periodontitis and monitoring the response to periodontal treatment (Stathopoulou et al., 2015; Cafiero et al., 2021). The relevance of these inflammatory mediators is supported by animal and human studies which reported that these biomarkers were significantly increased during periodontitis in relation to healthy periodontium (Shimada et al., 2010; Mohammad and Aziz, 2018; Leira et al., 2020; Khuda et al., 2021), and the levels of these biomarkers directly correlated with disease severity and alveolar bone loss (Leira et al., 2020; Koppolu et al., 2021; Luchian et al., 2022). The severity of periodontal disease has been associated with reduced serum levels of the anti-inflammatory cytokine IL-10 in contrast to an increase in inflammatory biomarkers (Leira et al., 2020).

The present study also found a reduction in inflammatory biomarkers and an elevation in anti-inflammatory cytokines in the three treatment groups from baseline to 2, 4, and 6 weeks. These results indicate that all three treatment modalities (CU, Tet, and SRP groups) were effective in reducing the inflammatory response in rats subjected to ligature-induced periodontitis and inoculation with *P. gingivalis*, with the Tet and Cu groups showing better improvement than the SRP group.

**Figure 5:**
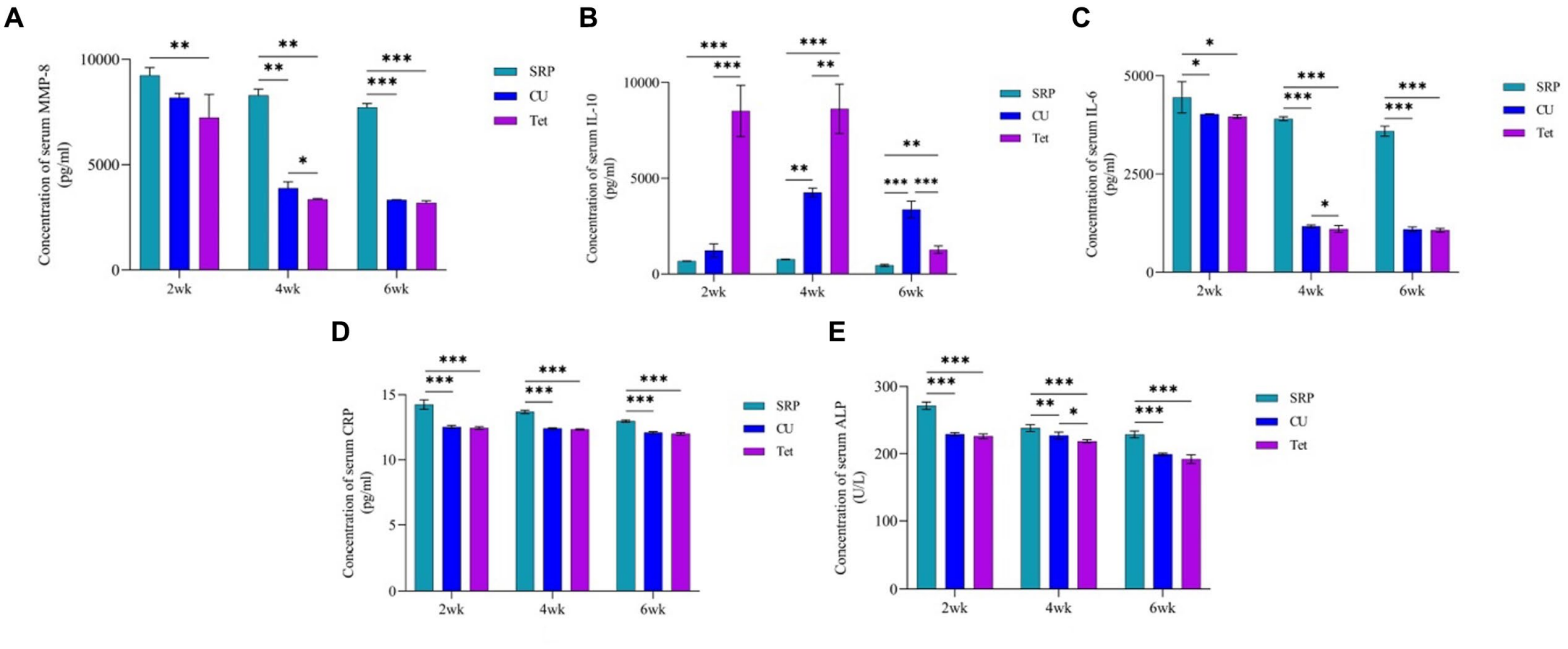
Comparison between the three studied treatment groups at 2, 4, and 6 weeks using one-way ANOVA with multiple group comparison using Tukey’s *post hoc* test for **(A)**: MMP-8, **(B)**: IL-10, **(C)**: IL-6, **(D)**: CRP, and **(E)**: ALP. CU, *Curcumin*; Tet, tetracycline; SRP, scaling and root planing; MMP, matrix metalloproteinase; IL-10, interlukine10; CRP, C-reactive protein; ALP, alkaline phosphatase enzyme; *p*, probability. *Significant, **Highly significant, ***Very highly significant.

On the other hand, the main differences (main improvements) in IL10, MM-8, IL-6, CRP, and ALP at the baseline time points in the EP group and the CU, Tet, and SRP groups are presented in Figure 4. The results showed that all tested biomarkers were highest in the Tet group, followed by the CU group, and lowest in the SRP group. Figure 5 presents the mean differences between baseline (0 days) in the EP group and the different treatment time points in the CU, Tet, and SRP groups. The main improvements in the blood levels of MMP-8, IL-6, IL-10, CRP, and ALP from 0 days to 2 weeks, from 0 days to 4 weeks, and from 0 days to 6 weeks were highest in the Tet group, followed by the CU group, and lowest in the SRP group.

The levels of IL-6, MMP-8, IL10, CRP, and ALP in the three treatment groups are presented in Figure 5. After 2, 4, and 6 weeks of periodontal therapy, the results revealed significant differences among the three groups. After 2 and 6 weeks of therapy, there were no significant changes in the reduction of IL-6, MMP-8, CRP, or ALP levels between the CU and Tet groups; however, there was a significant difference between the two groups at 4 weeks. Regarding IL-10, no significant differences were found between the CU and Tet groups after 2, 4 and 6 weeks of therapy. However, significant differences at different time points were found when the CU, Tet, and SRP groups were compared except for the levels of MMP-8 and IL-6 at 2 weeks.

In line with previous studies (Shimada et al., 2010; Mohammad and Aziz, 2018; Leira et al., 2020; Khuda et al., 2021; Koppolu et al., 2021; Luchian et al., 2022), the results of the present study showed that the EP group had significantly higher serum levels of IL-6, CRP, MMP-8, and ALP and lower levels of IL-10 than the control group confirming that the experimental model used was able to induce experimental periodontitis. This may be explained by the fact that the host’s immune inflammatory response to bacteria and bacterial products produces various proinflammatory mediators and cytokines as well as several tissue-degrading enzymes such as MMP that cause periodontal soft and hard tissue destruction (Meyle et al., 2017), and these inflammatory biomarkers will enter the circulation (Gu and Ryan, 2009). In contrast, some studies have reported the opposite of the current study; (Virtanen et al., 2017; Escobar et al., 2018), factors such as duration and the method of inducing periodontitis might explain these discrepancies in the results.

The anti-inflammatory properties of both gels when applied locally for 7 days and continued for 6 weeks after the last application of *CU* and Tet gels. These long duration effects can be explained by the bioadhesive property of *Curcumin*, which results in better retention in the periodontal pocket (Anuradha et al., 2015). Tetracycline retained the necessary drug concentration at the desired place and then being excreted in the active form for extended durations as it adheres to the root surfaces (Anuradha et al., 2015). In addition, Tetracycline could significantly inhibit collagenase activity by binding to the Ca^2+^ or Zn^2+^ (cations) required for the activation of MMPs (Marcaccini et al., 2010; Tilakaratne and Soory, 2014). These results are in accordance with previous studies (Rodrigues et al., 2004; Cazalis et al., 2009; Gu et al., 2011).

Although treatment with Tetracycline showed significant improvement in inflammatory biomarkers, the development of resistance genes in the subgingival microbiota following local application limits its use as an adjunct to periodontal therapy (Rodrigues et al., 2004; Arredondo et al., 2020). Therefore, it is preferable to choose a natural treatment for periodontal disease rather than chemotherapeutic drugs, particularly those with identical features and few adverse effects. *Curcumin* was therefore employed in the current study because it is entirely natural, safe, and harmless (Asher and Spelman, 2013; Hosseini and Hosseinzadeh, 2018; Soleimani et al., 2018), with anti-inflammatory and immunomodulatory characteristics (Kunnumakkara et al., 2017). Additionally, because CU contains the same β-diketone zinc-binding moiety as Tetracycline, it has similar anti-inflammatory effects to that of Tetracycline in terms of MMP inhibition and reduction of inflammatory mediators (Wang et al., 2019).

Similar to the current study, previous research has examined the impact of local application of *Curcumin* gel on decreasing serum levels of IL-6 and CRP in rats with experimentally induced periodontitis, and findings have shown that the *Curcumin*-treated group have lower levels of these biomarkers than those of the control group (Zhou et al., 2013; Gorabi et al., 2022). In our previous work, we have shown by histopathological examination that the adjunctive application of CU gel is capable of reduce inflammation and alveolar bone resorption. Further, the results suggested that CU gel has potential osteogenesis and healing effects (Mohammad et al., 2022).

In contrast, IL-10 has been reported to be involved in the pathogenesis of periodontitis, as it downregulates the immune response (Hu et al., 2009). In the current study, the reduction of inflammatory biomarkers in rats that received treatment with *Curcumin* was accompanied by an increase in IL-10 levels. This result is in line with previous studies reporting that *Curcumin* could enhance the production of IL-10, an anti-inflammatory cytokine (Mollazadeh et al., 2019).

The effects of *Curcumin* in the treatment of periodontal disease can be explained by its anti-inflammatory properties. For example, *Curcumin* has been reported to downregulate NF-κB (Banik et al., 2017), and expression of cell adhesion molecules (such as intercellular adhesion molecule 1, vascular adhesion molecule 1) and E selectin. Inflammatory cytokines, however, have been shown to upregulate expression of these molecules (Kawasaki et al., 2015). Moreover, the inhibitory effects of *Curcumin* on proinflammatory cytokines such as IL-6, cell migration through chemokines receptors and LPS-induced COX-2 expression are major factors in the pathogenesis of periodontal disease (Livada et al., 2017; Guzmán-Flores et al., 2023), and alveolar bone resorption might explain the beneficial effects of *Curcumin* as an adjunct to periodontal treatment (Hong et al., 2004).

*Curcumin* also inhibits the expression of inducible nitric oxide synthase (iNOS) enzyme by inflammatory and epithelial cells, which may play a role in periodontal inflammation (Nakatake et al., 2017; Wang et al., 2019). On the other hand, it has been reported *Curcumin* molecule bind to CXCL8 chemokine and inhibit ferroptosis in ligature-induced periodontal-diseased mice, thus, play a pivotal role in treatment of periodontitis (Huang et al., 2023; Wang et al., 2023). Finally, *Curcumin* inhibits the JAK/STAT signaling pathway and phosphorylation of p38 MAPK, thus reducing the expression of iNOS, COX-2, monocyte chemoattractant protein-1, and intercellular adhesion molecule-1 during inflammatory periodontal disease (Nakatake et al., 2017; Wang et al., 2019).

The application of natural *Curcumin* in clinical settings has major limitations, such as low systemic bioavailability due to a poor absorption rate in the gastrointestinal tract, short plasma half-life (Anand et al., 2007) and rapid metabolism following oral administration (Yang et al., 2007). To overcome these limitations, the current study utilized the local delivery of *Curcumin* as a gel over a systemic approach because of the bioadhesive property of *Curcumin*, resulting in better retention in the periodontal pocket.

## Conclusion

The results of the current study suggest that adjunctive local application of *Curcumin* to SRP has anti-inflammatory effects comparable to those of tetracycline for the treatment of EP in rats. *Curcumin* has a comparable effect on the reduction of inflammatory biomarkers (MMP-8, IL-6, CRP, and ALP) and the elevation of anti-inflammatory biomarkers (IL-10) to those of tetracycline following 6 weeks of application. Thus, *Curcumin* is a promising alternative to antibiotics in periodontal therapy.

## Data availability statement

The raw data supporting the conclusions of this article will be made available by the authors, without undue reservation.

## Ethics statement

The animal study was approved by Ethic Committee, College of Dentistry, Hawler Medical University. The study was conducted in accordance with the local legislation and institutional requirements.

## Author contributions

CM: Conceptualization, Methodology, Supervision, Writing – original draft. KA: Methodology, Writing – original draft, Data curation, Investigation, Resources. AS: Conceptualization, Project administration, Validation, Visualization, Writing – review & editing. SG: Validation, Visualization, Writing – review & editing, Formal analysis.
